# Impact of residual symptoms on bipolar disorder patient’s functioning

**DOI:** 10.1192/j.eurpsy.2026.11206

**Published:** 2026-07-31

**Authors:** R. Kammoun, H. Hsine, H. Ghabi, H. Nefzi, M. Karoui, F. Ellouze

**Affiliations:** 1 Psychiatry G, Razi Hospital, Manouba, Tunisia; 2 Psychiatry, La Métairie Clinic, Nyon, Switzerland

## Abstract

**Introduction:**

Bipolar disorder has been concidered as cyclical, but recent studies show some patients remain symptomatic between episodes.

**Objectives:**

This study aimed to identify residual symptoms in remitted bipolar I patients and explore the factors associated with these inter-episodic symptoms.

**Methods:**

This descriptive, cross-sectional, case-control, c comparative study involved 62 remitted bipolar I patients. Evaluation was carried out through a semi-structured questionnaire with the Bipolar Depression Rating Scale (BDRS), Young Mania Rating Scale (YMRS), Functioning Assessment Short Test (FAST), Arizona Sexual Experiences Scale (ASEX), and Pittsburgh Sleep Quality Index (PSQI). Patients were divided into two groups based on the presence or absence of residual symptoms.

**Results:**

Sixty-one of the patients had residual symptoms (Group 1) and sixty-one patients were free of residual symptoms (Group 2). In the first group, 15 patients had residual depressive symptoms (48%), 11 had residual manic symptoms (35%), 7 had impaired functioning (22%), 12 had sexual dysfunction (39%) and 25 had sleep disorders (78%). The residual symptoms that were significantly more important in Group 1 in comparison to Group 2 were: depressive symptoms (p=0.003), manic symptoms (p=0.005), impaired functioning (p=0.002) and sleep disturbances (p=0.000). Group 1 patients were significantly less educated (p=0.034) and less employed (p=0.032) compared to group 2 patients. They also had significantly more family history of psychiatric disorders and suicide attempts (p=0.038; p=0.039), more personal history of suicide attempts (p=0.012) and a higher number of hospitalizations (p=0.003).

**Conclusions:**

Residual symptoms are common in bipolar disorder and linked to poor prognosis, necessitating appropriate management.

**Disclosure of Interest:**

None Declared

